# Supply-demand matching of medical services at a city level under the background of hierarchical diagnosis and treatment - based on Didi Chuxing Data in Haikou, China

**DOI:** 10.1186/s12913-022-07762-4

**Published:** 2022-03-17

**Authors:** Haiyan Shao, Cheng Jin, Jing Xu, Yexi Zhong, Bing Xu

**Affiliations:** 1 School of Geography Science, Nanjing Normal University, Nanjing, 210023 People’s Republic of China; 2 Jiangsu Center for Collaborative Innovation in Geographical Information Resource Development and Application, Nanjing, 210023 People’s Republic of China; 3 Tourism and Social Administration College, Nanjing Xiaozhuang University, Nanjing, 211171 People’s Republic of China; 4 School of Geography and Environment, Jiangxi Normal University, Nanchang, 330022 People’s Republic of China; 5 Second Clinical Medical College, Guangzhou University of Chinese Medicine, Guangzhou, 510405 People’s Republic of China

**Keywords:** Hierarchical diagnosis and treatment, Medical supply and demand, Two-step floating catchment area method, Didi Chuxing data, Haikou City

## Abstract

**Background:**

Implementation of the Healthy China Strategy and the hierarchical diagnosis and treatment system has injected new vitality into medical services. Given the insufficient supply of medical services and increasing demand for medical treatment, exploring the supply-demand pattern of medical services has become an urgent theoretical and practical problem to be solved. The equity of healthcare facilities has received widespread attention, but due to limited data, there is little research on the supply-demand pattern of medical services. This study focuses on evaluating the supply-demand matching pattern of medical services at different levels in Haikou City with big geographic data and promoting the realization of a balance between medical supply and demand.

**Methods:**

This study utilizes spatial data of medical institutions, Didi Chuxing Data, and population density data. Firstly, use the two-step floating catchment area method and GIS spatial analysis to explore characteristics of the supply-demand patterns of medical services at different levels in Haikou. Secondly, we mine residents’ demand for medical treatment based on Didi Chuxing Data. Then combined with population density data, divide supply-demand matching of medical institutions into four types. Finally, propose optimization strategies for the problems.

**Results:**

The accessibility pattern of high-level medical institutions in Haikou presents high in the north and low in the south. The accessibility pattern of low-level medical institutions is the opposite. High-level medical institutions have a strong demand for medical treatment, which is less hampered by distance. The healthcare demand of low-level medical institutions is small, and they mainly are medium- and short-distance medical travel. The types of medical services at different levels are mainly “low supply - low demand” and “high supply - low demand” types.

**Conclusions:**

Medical services at different levels in Haikou are mainly in supply-demand imbalance. Therefore, we put forward optimization strategies to promote the equity of primary medical services, such as propelling the establishment and improvement of the hierarchical diagnosis and treatment system, building a new model of medical and health service supply, and strengthening balanced coverage of primary medical institutions. The mining of big geographic data is beneficial to alleviate the mismatch between medical supply and demand, although the data and methods need to be improved.

## Background

Over the past 40 years of reform and opening-up, China has made remarkable achievements in the reform and development of the health sector [1]. However, rapid urbanization, changes in the disease spectrum, and lifestyle have brought many adverse effects, such as the insufficient supply of medical services and rising demand for medical treatment [2–4]. In recent years, the state has issued a series of policy opinions to guide the rational allocation of medical resources and equal development of primary medical and health services [5, 6]. In September 2015, the General Office of the State Council of the People’s Republic of China issued Guiding Opinions on Promoting the Construction of Hierarchical Diagnosis and Treatment System, requiring the establishment of a hierarchical diagnosis system to guide the sinking of high-quality medical resources and effectively promote the equity and accessibility of primary medical and health services. The Outline of Healthy China 2030 Plan pointed out that we should improve the medical and health services system from both the supply and demand sides in October 2016. We can see that the research on supply-demand matching of medical services has a strong practical need and important theoretical significance.

As one of the core topics of health geography research, previous research on medical services has mainly focused on three aspects: the spatial layout and optimal allocation of medical service facilities, the accessibility and equity of medical facilities, and the supply-demand matching of medical services [7–9]. In research on spatial layout and optimal allocation, scholars have discussed the location selection of medical service facilities based on location theory, spatial interaction theory, and central place theory in the early days [10]. The connotation analysis of fairness and efficiency in the location selection of medical service facilities and the research on the location selection method of medical service facilities based on fairness are gradually carried out. With the rapid combination of location-allocation models and spatial analysis methods, the focus of related research has shifted from more emphasis on the theoretical model of location selection of medical service facilities to the simultaneous development of the theory and practical application of spatial location selection and layout optimization of medical service facilities [11]. In the field of accessibility and equity research, based on the accessibility theory [12–14], scholars mostly use the two-step floating catchment area method, the Gaussian two-step floating catchment area method, the accessibility of gravity coefficient, the improved potential model, and other methods to measure the spatial accessibility of medical service facilities [15–18]. And from the aspects of method improvement [19–21], supply evaluation [22, 23], transportation modes [24], spatial effects [25], influencing factors [26], and other multiple dimensions to expand the connotation and extension of medical accessibility research. In supply and demand matching research, scholars have divided the medical service pattern into supply and demand patterns based on the theory of supply and demand [27]. In terms of supply and demand identification, some scholars rely on the medical staff and the population to identify areas where medical resources are scarce [28]. In addition, some scholars reflect the intensity of medical demand through data such as socio-demographic, economic, and hospital levels and use an improved gravitational model to identify medical shortage areas [29]. With the gradual implementation and deepening of the Healthy China Strategy and the hierarchical diagnosis and treatment system, the research on hierarchical medical services has increasingly become a hot spot in the academic world [30]. At present, the medical services research system has made a lot of beneficial explorations in medical supply, which also provides some reference for this paper. However, the empirical research on the characteristics of hierarchical medical services supply and demand in the existing literature is still in the initial exploratory stage.

The hierarchical diagnosis and treatment system is an important measure to promote the effective utilization of medical resources and the critical content of medical and health system reform. It is based on the basic principles of “first diagnosis at primary level, two-way referral, separation of acute and chronic treatment, and upper and lower linkages. “ It is graded according to the severity of disease and treatment difficulty. Residents follow the hierarchical treatment and referral procedures of “township health centers and community health service centers (primary-level medical and health institutions) - first-level designated medical institutions - second-level designated medical institutions - third-level designated medical institutions. [31]“ As one of China’s rapidly urbanized areas, Haikou City still has unbalanced allocation and spatial polarization in the construction of medical service facilities, which leads to problems such as lack of social equity and low utilization efficiency of medical resources [32]. In recent years, Haikou has actively established a new model of hierarchical diagnosis and treatment and continuously improved the service level of medical and health institutions at different levels. Therefore, this paper takes Haikou City as the research area, based on Didi Chuxing Data, uses two-step floating catchment area method and spatial analyst method to explore the supply-demand matching pattern of medical services, and then proposes optimization strategies to provide a reference for the rational allocation of medical resources and the equal development of medical services.

The possible marginal contributions of this paper are as follows: (1) The update of the research perspective. The hierarchical diagnosis and treatment system provides new ideas for solving the structural contradictions of medical resources, improving the efficiency of medical services, and optimizing medical service models. Although scholars have started to pay attention to the spatial accessibility of hierarchical medical service facilities, there is still little medical service research focusing on the background of hierarchical diagnosis and treatment. (2) Improvement of research ideas. At present, relevant literature has focused on the supply-side research of medical services. With the increasingly acute contradiction between the supply and demand for medical services, research considering both the supply and demand sides is spawned. (3) Innovation of research data. Unlike the existing literature mainly uses accessibility data and population density data to measure supply and demand, this paper considers the static and hierarchical homogeneity of population density data and the time continuity of medical behaviors. We obtain medical travel based on Didi Chuxing Data to measure the supply-demand matching pattern of medical services more accurately.

## Methods

### Two-step floating catchment area method

Spatial accessibility is one of the essential indicators to evaluate the supply level of public facilities services [33]. It focuses on the distance between supply and demand points or the transportation cost generated on the possibility of consuming services [34]. The two-step floating catchment method (2SFCA) and the gravity model method widely evaluate spatial accessibility. Based on the same theoretical framework, both comprehensively consider the impact of the supply scale, demand scale, and distance relationship between supply and demand for accessibility. The difference lies in the treatment of distance factors. The gravity model method adopts the continuous distance attenuation function, which considers the characteristics of facility service capacity attenuation with distance. Still, it does not limit the effective search radius of the facility. The two-step floating catchment area method uses dichotomy to deal with distance attenuation. The same accessibility is within the search radius threshold, but it is entirely unreachable outside the search radius [35, 36]. Under the condition that the total amount and layout of medical resources and facilities are relatively stable, the distance factor plays a significant role in residents’ choice of medical travel. It is also a key consideration factor for geographers to evaluate medical fairness. The two-step floating catchment method (2SFCA) is based on chance accumulation, which has been widely used in the accessibility measurement of healthcare facilities in recent years. The advantage is that, on the one hand, the interrelationship between the supply and demand of the research object is considered; on the other hand, the characteristics of the facility service capacity decay with distance are considered, and the concept of effective search radius is added. The average value of the shortest travel distance from administrative villages (communities) to medical and health institutions is the effective search radius. Medical travel activities within this radius are relatively active, conforming to the law of human travel behavior. Therefore, this paper adopts the two-step floating catchment method to measure the supply pattern of medical services.

Firstly, determine the supply-demand ratio within the service threshold (d_0_) of the supply points (medical and health institutions). Secondly, calculate the accessibility of residents to seek medical services, that is, calculate the number of medical and health institutions within reach of each administrative village (community). These supply-demand ratios of medical and health institutions are summed to obtain the medical accessibility of each administrative village (community). Since the spatial accessibility varies with different service thresholds, we take the average of the shortest travel distances from all administrative villages (communities) to medical and health institutions like the medical service thresholds at different levels. The service thresholds of tertiary hospitals, secondary hospitals, primary hospitals, and primary medical institutions are 17.14 km, 16.73 km, 19.25 km, and 21.73 km, respectively. Only suppliers and demanders within the threshold range can have a spatial correlation.

In the first step, for each medical and health institution j, search all administrative villages (communities) k within the distance threshold from j, and calculate the supply-demand ratio R_j_ of each medical and health institution:

1 $${\mathrm{R}}_{\mathrm{j}}=\frac{{\mathrm{S}}_{\mathrm{j}}}{\sum_{\mathrm{k}\in \left\{{\mathrm{d}}_{\mathrm{k}\mathrm{j}}\le {\mathrm{d}}_0\right\}}{\mathrm{D}}_{\mathrm{k}}}$$

Where: d_kj_ is the distance between administrative villages (communities) k and medical and health institutions j; D_k_ is the average population density of administrative villages (communities) in the search area; S_j_ is the number of medical and health personnel at point j, which is determined according to the Hospital Classification Management Standards, that is, the total supply of medical services.

The second step is to search the number of medical institutions within the service threshold (d_0_) for each village-level (community-level) residential area i. And add the supply-demand ratio R_j_ of all medical institutions to obtain the accessibility ${\mathrm A}_{\mathrm i}^{\mathrm F}$ of administrative villages (communities) i:

2 $${\mathrm{A}}_{\mathrm{i}}^{\mathrm{F}}=\sum_{\mathrm{j}\in \left\{{\mathrm{d}}_{\mathrm{i}\mathrm{j}}\le {\mathrm{d}}_0\right\}}{\mathrm{R}}_{\mathrm{j}}=\sum_{\mathrm{j}\in \left\{{\mathrm{d}}_{\mathrm{i}\mathrm{j}}\le {\mathrm{d}}_0\right\}}\left[\frac{{\mathrm{S}}_{\mathrm{j}}}{\sum_{\mathrm{k}\in \left\{{\mathrm{d}}_{\mathrm{k}\mathrm{j}}\le {\mathrm{d}}_0\right\}}{\mathrm{D}}_{\mathrm{k}}}\right]$$

Where: d_ij_ is the distance between village-level (community-level) settlements i and j; R_j_ is the supply-demand ratio of medical and health institution j in the i search area (d_ij_ ≤ d_0_) centered on the administrative villages (communities). The larger ${\mathrm A}_{\mathrm i}^{\mathrm F}$, the better accessibility.

### Data source and processing

**Fig. 1:**
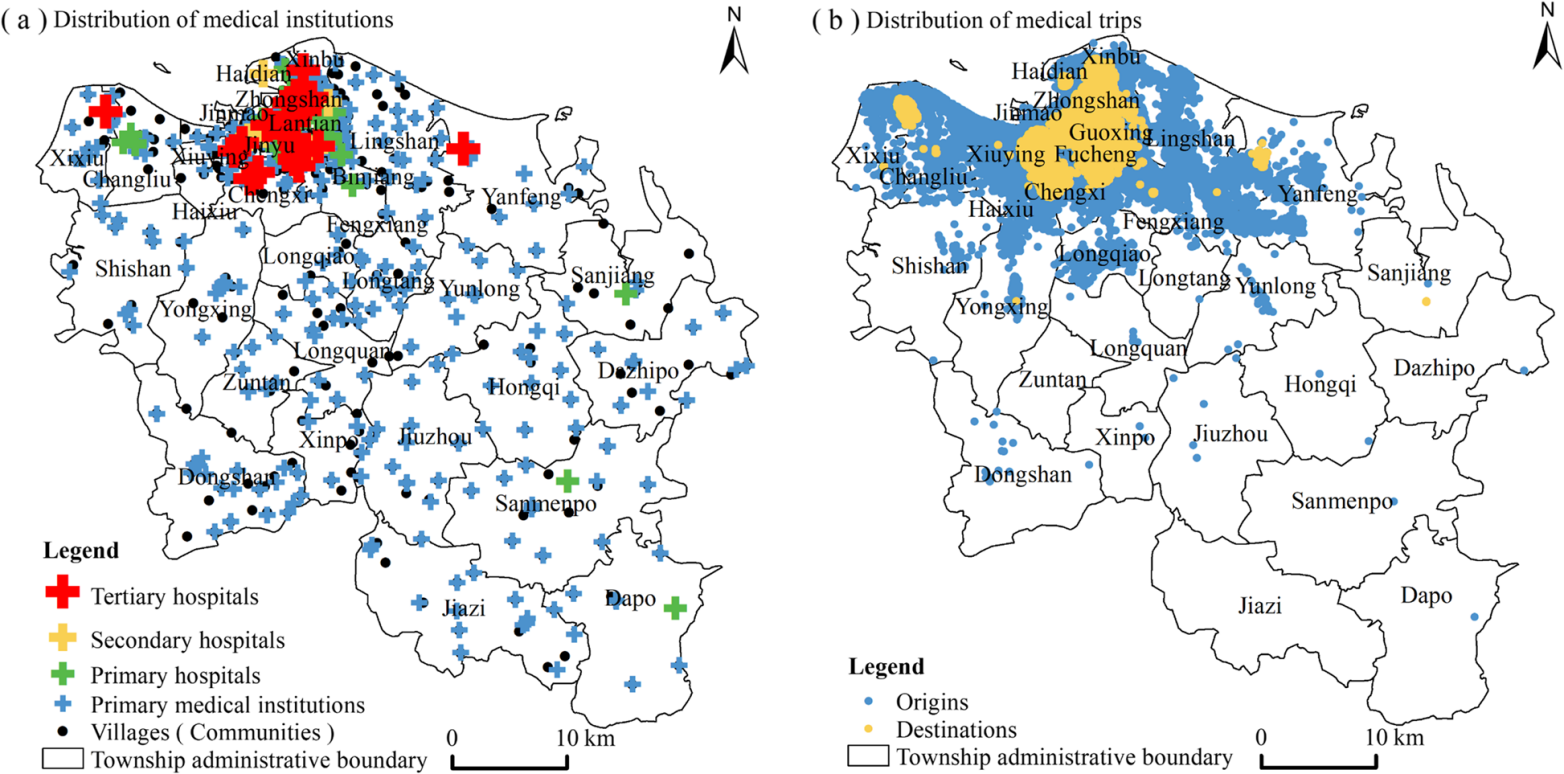
Distribution of medical institutions and medical trips in Haikou City

The data used in this paper mainly include spatial data of medical institutions and administrative villages (communities), population density data, and Didi Chuxing Data. The spatial attribute data of medical and health institutions in the study area are obtained through Python language and the open API interface of the Baidu map. It is sifted in combination with the list of medical and health institutions in Haikou City issued by the Hainan Health Commission. The grade of each medical and health institution is determined according to the national medical institution query service provided by the National Health Commission. The number of tertiary hospitals, secondary hospitals, primary hospitals, and primary medical institutions is 22, 7, 29, and 351. The data of administrative villages (communities) are obtained based on Haikou Statistical Yearbook 2018. By picking up the longitude and latitude coordinates of each residential area for spatial visualization (Fig. 1), a total of 429 residential areas are obtained. The population data of Haikou in 2017 is derived from WorldPop population density data, with a spatial resolution of 1 km × 1 km. Owing to the deviation of the data, the average population density of the statistical unit grid based on the source data is used as the basis for analysis. Didi Chuxing Data comes from the “Gaia” data open plan (https://gaia.didichuxing.com), including attribute information such as longitude and latitude of the origin and destination of the order, order ID, timeliness of the order. Select order data from September 2017. In the process of data pretreatment, identify the only case with the order ID, screen the real-time orders by order timeliness. With the consideration of the service level for medical institutions at different levels, the buffer distance of medical and health institutions was divided into 1000 m, 500 m, 100 m, and 50 m in ascending order. Thus, there were 1093,697 medical flows in tertiary hospitals, 85,488 in secondary hospitals, 29,617 in primary hospitals, and 16,807 in primary medical institutions. Meanwhile, Haikou City was divided into a 1 km × 1 km hexagonal grid as a statistical analysis unit.

## Results

### Supply patterns of medical services at different levels

**Fig. 2:**
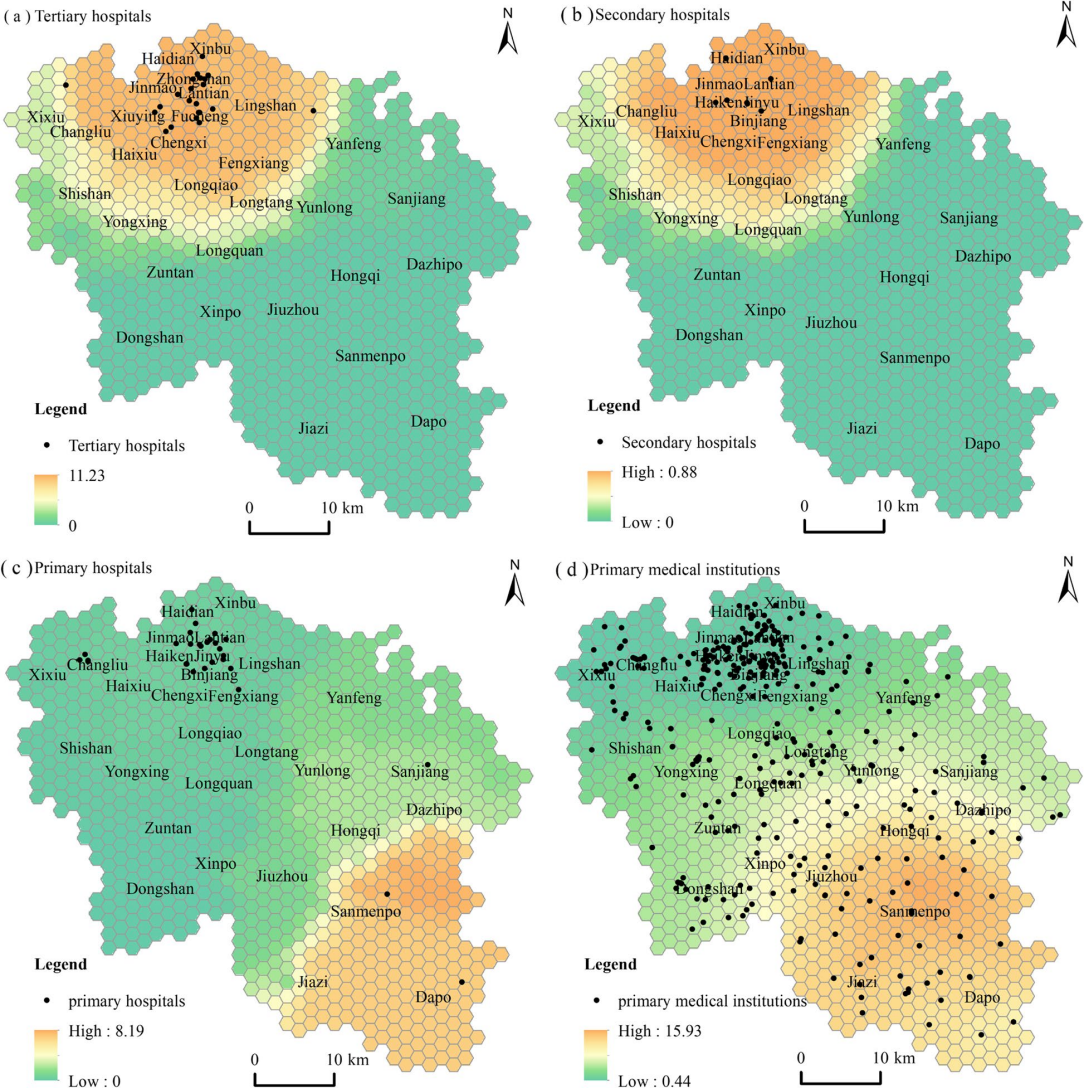
Accessibility patterns of medical services at different levels

The two-step floating catchment area method is used to mine the supply pattern of medical services. The spatial accessibility of medical and health institutions at different levels in Haikou City is calculated, as shown in Fig. 2.

Specifically, tertiary and secondary hospitals are concentrated in the downtown area of ​​Haikou. They have a relatively similar spatial accessibility pattern, characterized by a gradual decrease from north to south. The medical services supply level of Jinmao Street, Haiken Street, Binjiang Street, Haixiu Street, Fengxiang Street, Bailong Street, Haidian Street, Xinbu Street, Lingshan Town, Chengxi Town, Haixiu Town, Changliu Town, and Longqiao Town is good. The supply of medical services in Dongshan Town, Xinpo Town, Jiuzhou Town, Hongqi Town, Dazhipo Town, Sanmenpo Town, Jiazi Town, and Dapo Town is relatively poor. As can be seen from the figure, the supply of high-level medical and health institutions is mainly oriented to the central urban area in the north, with a small range of supply services. The southeast half of the city is a weak area of medical service supply, and most cities and towns cannot enjoy the supply services of high-level medical institutions. The reason is that the terrain in the north of Haikou is flat, the road network is dense, and the population is concentrated. The superior natural and cultural environment attracts the concentrated distribution of high-level medical and health institutions, making it more convenient for residents in the north to obtain medical services. Topography, the number, location, and scale of surrounding medical facilities have a significant boundary effect, resulting in poor spatial accessibility of high-level medical institutions.

The accessibility of primary hospitals and primary medical institutions is contrary to that of high-level medical and health institutions, showing the characteristics of weakening from south to north. The high-value accessibility areas of primary hospitals are distributed in Sanmenpo Town, Jiazi town, and Dapo Town, and the low value of accessibility is located in the west. Primary medical institutions are widely distributed. Sanmenpo Town, Jiazi Town, Dapo Town, Jiuzhou Town, Hongqi Town, Dazhipo Town are high-value accessibility areas in the south. Dongshan Town, Xinpo Town, Yunlong Town, and Sanjiang Town are high and low accessibility transition areas in the middle, and the north is a low accessibility value area. In summary, the accessibility patterns of medical and health institutions at different levels present a core edge structure, and there are obvious spatial differences.

### The characteristics of medical demands of residents at different levels

**Fig. 3:**
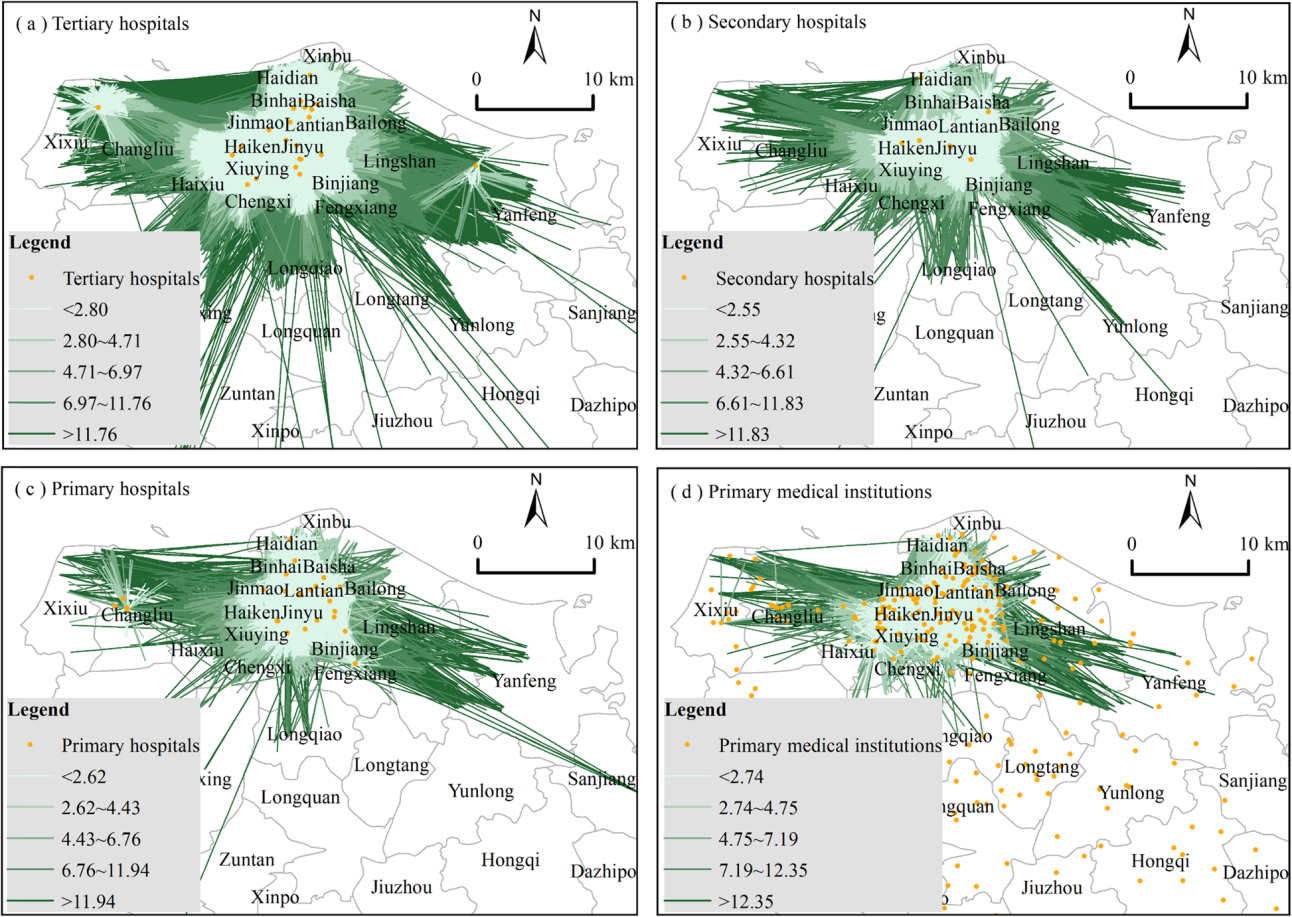
Distribution of medical demand based on Didi Chuxing

Considering the stagnation of population density data, we obtain medical travel orders of medical institutions at different levels based on Didi Chuxing Data to objectively and genuinely represent the spatial differences in medical demand. With the XY to Line tool of ArcGIS, the travel characteristics of medical institutions at different levels in Haikou City are drawn, and medical flows are divided into five classes according to the distance attribute by using the natural break method, as shown in Fig. 3.

On the whole, medical flows in Haikou City present apparent polarization centripetal characteristics, mainly distributed in the northern region, and overlaps with the economic development level and population density of Haikou City to a certain extent. Specifically, tertiary hospitals have strong demand for medical treatment. The tertiary hospitals distributed in the central urban area gather most of the demand for medical treatment, reflecting the polarization effect of high-level hospitals. The number of medium- and long-distance medical flows is extensive, with 22,851 medical flows with a distance of more than 11.76 km (Table 1). The Hainan Provincial Cancer Hospital in the west and the Fifth People’s Hospital of Hainan Province (Guilinyang District) in the east have dispersed part of the medium- and short-distance medical demands. The demand for medical treatment in secondary hospitals is relatively strong. The long-distance demand areas are mainly distributed in Yanfeng Town, Longqiao Town, Chengxi Town, Changliu Town, and Xixiu Town. Primary hospitals and primary medical institutions have small demand for medical treatment, and the behavior is dominated by medium- and short-distance medical treatment. High-level hospitals have higher attractiveness, while lower-level hospitals have less attractiveness, which leads to increased pressure on medical demand.

**Table 1:** Statistics on the number of medical flows at different levels

Distance level	Tertiary hospital		Secondary hospital		Primary hospital		Primary medical institution	
	Medical flow (Bar)	Flow ratio(%)	Medical flow (Bar)	Flow ratio(%)	Medical flow (Bar)	Flow ratio(%)	Medical flow (Bar)	Flow ratio(%)
First level	337,302	30.84	27,879	32.61	9187	31.02	5658	33.66
Second Level	303,706	27.77	28,724	33.60	10,188	34.40	5890	35.04
Third level	360,027	32.92	19,880	23.25	7247	24.47	3558	21.17
Fourth level	69,811	6.38	7293	8.53	2232	7.54	1408	8.38
Fifth level	22,851	2.09	1712	2.00	763	2.58	293	1.74

### Optimization of supply and demand of medical services at different levels

#### Supply-demand matching pattern of medical services at different levels

**Fig. 4:**
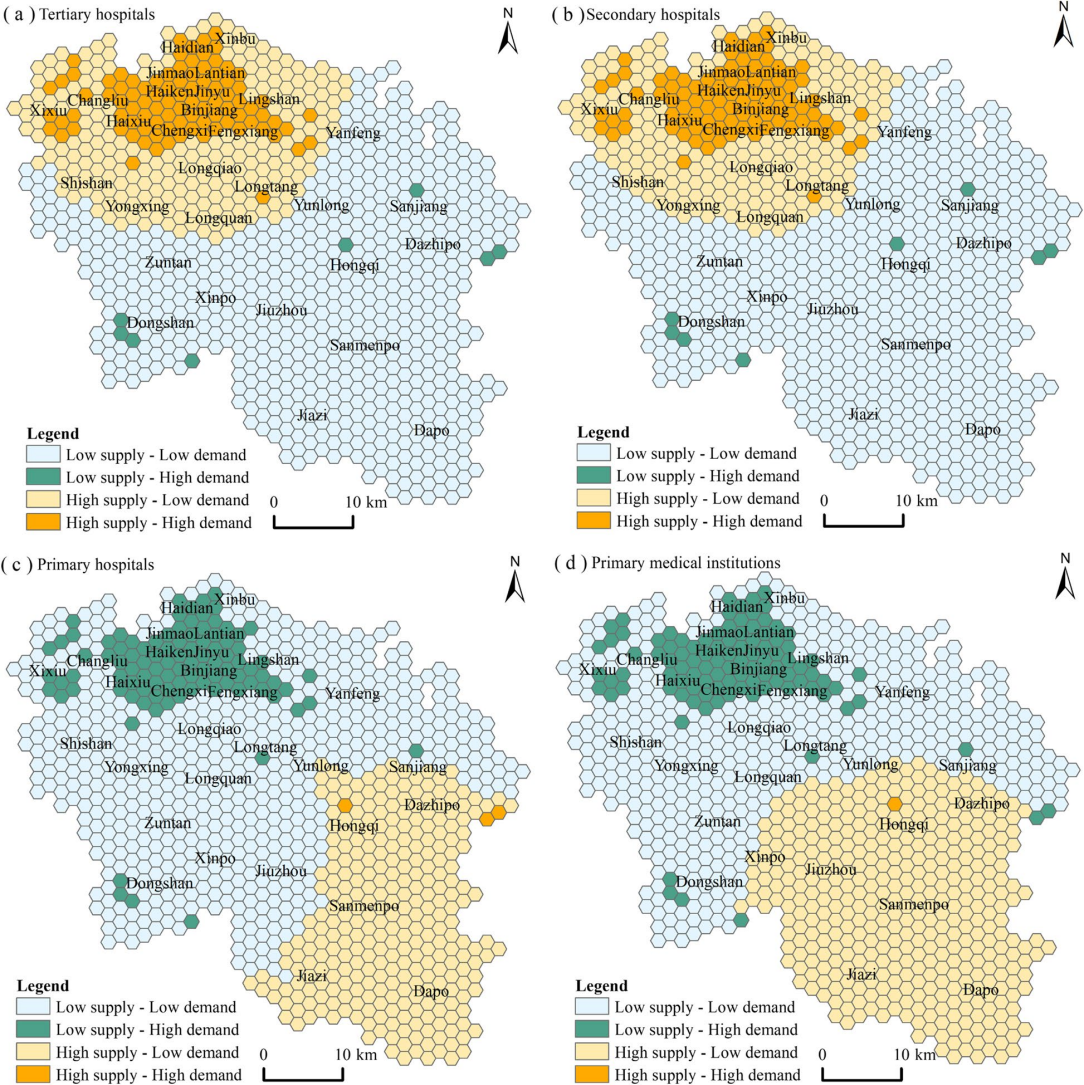
Supply-demand matching pattern of medical services at different levels

The spatial accessibility of medical institutions at different levels represents the supply pattern of medical services. Combined with the population density data of Haikou City in 2017 and the order data of Didi Chuxing, a weight of 0.5 is assigned to determine the demand pattern of medical services at different levels. Thereby, we obtain the supply-demand matching pattern of medical services in Haikou (Fig. 4).

In terms of quantity distribution, the matching types of medical services supply and demand at different levels are mainly “low supply - low demand” and “high supply - low demand.” (Table 2). There are a few “high supply - high demand” and “low supply - high demand” types. The supply and demand matching types of tertiary and secondary hospitals are dominated by “low supply - low demand,” accounting for 66.32% and 67.97% of the regional grid. The “high supply - low demand” type followed, accounting for 23.20% and 21.36%, respectively. The number of “high supply - high demand” types is small, accounting for 9.65% and 9.86%, respectively. The “low supply - high demand” type is the least. The supply and demand matching types of primary hospitals and primary medical institutions are mainly “low supply - low demand, accounting for 59.55% and 48.56% respectively. The “high supply - low demand“ type followed, accounting for 29.98% and 40.86%, respectively. There are few types of “low supply - low demand,” accounting for 10.16% and 10.47%, respectively.

**Table 2:** Statistics on the supply-demand matching of medical services at different levels

Type	Tertiary hospital		Secondary hospital		Primary hospital		Primary medical institution	
	Amount (pcs)	Percentage (%)	Amount (pcs)	Percentage (%)	Amount (pcs)	Percentage (%)	Amount (pcs)	Percentage (%)
Low supply – Low demand	646	66.32	662	67.97	580	59.55	473	48.56
Low supply – High demand	8	0.82	8	0.82	99	10.16	102	10.47
High supply – Low demand	226	23.20	208	21.36	292	29.98	398	40.86
High supply – High demand	94	9.65	96	9.86	3	0.31	1	0.10

In terms of spatial distribution, the supply-demand patterns of medical services in tertiary and secondary hospitals have high similarities. The “high supply - high demand” types are mainly distributed in the main urban area of ​​Haikou City, and the “high supply - low demand” type is located outside the “high supply - high demand” type, showing a circular distribution. The “low supply - low demand” type is mainly distributed in the south, showing the characteristics of concentrated and contiguous. The “low supply - high demand” type is scattered in the central region. The “high supply - high demand” types of primary hospitals and primary medical institutions are distributed in isolation. The “high supply - low demand” types are mainly distributed in the southeast, the “low supply - low demand” types are distributed in the middle, and the “low supply - high demand” is distributed in the core urban areas. Therefore, it is urgent to solve the imbalance between the supply and demand of medical services at different levels in Haikou.

#### Optimization strategies of the supply-demand balance of medical services at different levels

Focusing on the spatial accessibility of medical and health institutions and residents’ medical demand is an effective way to solve the mismatch between the supply and demand of medical services. Based on the above analysis, there are many supply gaps in medical institutions at different levels in Haikou City. The spatial differentiation of medical demand is evident, with hot spots in the north and cold spots in the south. Medical services at different levels are mainly in a state of imbalance between supply and demand. Aiming at the supply-demand matching pattern of medical services at different levels in Haikou City, the following optimization strategies are proposed:

1. Promote the establishment and improvement of the hierarchical diagnosis and treatment system. Implementing the hierarchical diagnosis and treatment system is an essential task in China’s “14th Five-Year Plan” period to deepen the reform of the medical and health system, focusing on the coordination and integration of medical services at different levels. At this stage, high-level medical and health institutions are the first choice for residents to seek medical treatment. In contrast, low-level medical and health institutions are lack attraction to residents. Therefore, it is necessary to promote the establishment of the hierarchical diagnosis and treatment system with a clear division of labor, efficient collaboration, and complete functions. Under the premise of taking efficiency and equity into consideration, promote the expansion and upgrading of high-quality medical resources in the northern central area of ​​Haikou City. And enhance the supply level of medical services, add high-level hospitals in the central and southern regions to promote the balanced allocation of high-quality medical and health resources. Everyone can enjoy homogeneous diagnosis and treatment of acute and difficult diseases and specialized medical services. Set up more primary medical institutions in the supply blind area of low-level medical and health institutions to guide the rational flow of residents for medical treatment, and promote the formation of an orderly pattern of medical treatment. At the same time, clarify the functional positioning of medical institutions at different levels and promote differentiated medical facilities to meet residents’ multi-level and diversified medical demand.
2. Build a new model of medical and health service supply. As high-level hospitals cover a broader population, the over-concentrated layout will quickly lead to increased demand for medical care and excessive traffic congestion around them. In contrast, the medical service resources of low-level hospitals are primarily idle and inefficient in utilization. Therefore, it is necessary to improve multiple modes of labor division and collaboration, such as medical consortia and hospital groups, to enhance the overall effectiveness of the medical service system. On the one hand, according to the distribution of medical institutions, residents’ medical demand, population density, and other factors promote the reasonable sinking and balanced distribution of high-quality medical resources. Guide tertiary hospitals to gradually reduce general outpatient clinics and focus on developing the diagnosis and treatment of acute, severe, and intractable diseases. On the other hand, it gives play to the radiating and driving role of high-quality medical resources. Build a multi-level linkage medical service network and strengthen the sharing of medical help within the region to make up for the service shortcomings of low-level medical institutions and reduce residents’ cross-regional, long-distance and high-cost medical treatment.
3. Strengthen the balanced coverage of primary medical institutions. Haikou’s primary medical institutions are widely distributed, but the problem of “mismatch between supply and demand” of medical services is prominent. Therefore, it is necessary to focus on rural and primary levels, promote the equalization of essential medical public services, and gradually narrow the differences in medical service levels between urban and rural areas, regions, and populations. We should rationally allocate primary medical and health resources according to population density and service radius. Everyone can enjoy equal access to primary medical and health services to disperse the pressure of medical demand and accelerate the speed of medical treatment. It is employing government organization or purchase of services, the scientific layout of primary medical and health institutions, rational division of service areas, standardization construction, to realize full coverage of urban and rural residents. By building medical consortia, counterpart support, and multi-point practice of doctors, doctors in tertiary and secondary hospitals are encouraged to practice in primary medical and health institutions or make regular visits and rounds to improve primary service capacity. Promote the software and hardware capabilities of primary medical and health institutions, strengthen the primary medical service functions of township hospitals, and improve the medical service capabilities of emergency rescue, routine operations below level II, and pediatrics. The utilization efficiency of primary medical institutions in Haikou City is low, so it is urgent to strengthen the function positioning of primary medical services.

## Conclusion and discussion

### Conclusion

Based on the data of medical and health institutions and Didi Chuxing Order Data, this paper analyzes the supply pattern, demand characteristics, and supply-demand matching pattern of medical services in Haikou City in 2017, which can reveal not only the spatial differences between supply and demand of medical services but also perspective the new situations and problems of medical service development. The main conclusions are as follows:

1. The accessibility pattern of medical and health institutions at different levels in Haikou presents a core edge structure with noticeable spatial differences. The accessibility pattern of high-level medical institutions is characterized by a gradual decrease from north to south. The supply of medical services is mainly oriented to the central urban area in the north, with a small scope of service supply, and the southeast half is a weak area of medical service supply. The low-level medical institutions show the characteristics of weakening from south to north. There are many supply gaps in medical institutions at different levels.
2. The demand for medical treatment of different levels of medical institutions shows prominent polarized centripetal characteristics, mainly concentrated in the northern region, which overlaps with Haikou’s economic development level and population density to a certain extent. In contrast, the demand in the south is a mainly blank area. High-level medical institutions have a strong demand for medical treatment and are less affected by distance. Low-level medical institutions have a small demand for medical treatment, and they are mainly responsible for short- and medium-distance medical treatment.
3. The supply and demand types of medical services at different levels are mainly “low supply - low demand” and “high supply - low demand.”. The supply and demand patterns of high-level medical services have high similarities. The “high supply - high demand” types are mainly distributed in the main urban area of ​​Haikou City. The “high supply - low demand” types are distributed in a ring shape along with the “high supply - high demand” types, and “low supply - low demand” types are mainly distributed in the southeast. The “high supply - high demand” types of low-level medical institutions are distributed in isolation, the “high supply - low demand” types are mainly distributed in the east, the “low supply - low demand” types are distributed in the middle, and the “low supply - high demand” types are distributed in the core urban area. Medical services at different levels are mainly in a state of imbalance between supply and demand.
4. Aiming at the imbalance between supply and demand of medical services at different levels in Haikou City, it is proposed that we should promote the establishment and improvement of the hierarchical diagnosis and treatment system, build a new model of medical and health service supply, and strengthen the balanced coverage of primary medical institutions to promote the mismatch of the supply and demand of medical services at different levels to the balanced development of supply and demand.

### Discussion

Since the reform and opening-up, China’s urbanization process has been advancing rapidly, and the residents’ lifestyles have undergone drastic changes, resulting in a series of unbalanced development problems. Focusing on the supply level of medical institutions and the individual demand of residents for medical treatment and exploring the optimization strategy of medical service supply and demand is the critical way to solve the problem of medical service imbalance. This paper focuses on the background of hierarchical diagnosis and treatment. It takes hierarchical medical and health institutions as the research object, extending previous studies that only targeted a single type of research such as tertiary hospitals, general hospitals, urban medical facilities, rural medical resources, and emergency service facilities [37–39]. Previous related studies mostly started from the supply dimension and analyzed the spatial accessibility of medical service facilities, while the demand dimension analysis is relatively lacking. This paper discusses the supply-demand matching pattern of medical services, which supplements and enriches medical research on medical equity. In addition, previous studies have mainly used data such as social population, economy, number of doctors, and hospital level to identify medically saturated areas and deficient areas. This paper introduces traffic indicators to represent the demand for medical services using Didi Chuxing Order Data. Compared with conventional indicators, Didi Chuxing Order Data contains fine-grained spatial information to describe residents’ medical travel activities. Analyzing travel OD data makes it feasible to infer residents’ medical travel without violating privacy. Second, Didi Chuxing Order Data usually covers the entire city area and records location points in real-time. Therefore, compared with traditional statistics and survey data, Didi Chuxing Order Data has a shorter update cycle, broader spatial coverage, and larger observation scale. Third, while detailed and comprehensive survey data are often available in developed countries, they may not be available in other countries, such as China. Although some statistical data of medical resources can be found in some Chinese cities, they have somewhat limited spatial information related to the travel patterns of individual residents for medical-seeking activities. Therefore, to study the supply and demand matching of medical services in China from a spatial perspective, one should consider making full use of the available data at hand, such as Didi Chuxing Order Data. In conclusion, big geographic data provides the possibility for medical service demand mining. Based on Didi Chuxing Order Data, this paper conducts a supply-demand matching analysis. It is a beneficial attempt to solve the practical dilemma that population density cannot distinguish the difference of medical demand at different levels and avoids the one-sidedness of a single population density element. Based on the analysis of supply and demand patterns, it is found that medical services in Haikou are mainly in a state of imbalance between supply and demand. And relevant optimization strategies are proposed, which is beneficial for the government to deepen the reform of medical and health systems and promote the balanced development of medical services.

At present, the research on supply-demand matching of medical services, as a hot topic in health geography, is still in the stage of continuous improvement. This paper still has some shortcomings, especially limited by objective factors such as data. Firstly, this paper only discusses public medical and health institutions as the research object. Still, the overall medical and health service system covers a wide range, and more empirical research on medical facility data is still needed to test the conclusion of this paper. Secondly, with the improvement of the urban transportation system and the steady improvement of residents’ living standards, residents’ travel modes for medical treatment show a diversified trend. This paper only focuses on the online car-hailing travel modes. Although it does not hinder the study’s validity. There is an unavoidable omission in the demand collection of medical patients who use taxis, private cars, public transportation, and other modes of travel. Finally, this paper uses Didi Chuxing Data to characterize residents’ medical demands. Due to the limited time of data acquisition, only single-month data is selected for research. In the future, long-term data need to be used for further analysis. We can also combine it with mobile phone positioning data, taxi trajectory data, and other urban spatio-temporal big data mining medical service demands.

## Data Availability

The datasets used and/or analysed during the current study are not publicly available due to privacy and data protection concerns (open requirements) but can be made available from the corresponding author on reasonable request.
